# Ropivacaine infiltration analgesia of the drainage exit site enhanced analgesic effects after breast Cancer surgery: a randomized controlled trial

**DOI:** 10.1186/s12871-020-01175-8

**Published:** 2020-10-06

**Authors:** Baona Wang, Tao Yan, Xiangyi Kong, Li Sun, Hui Zheng, Guohua Zhang

**Affiliations:** 1 Department of Anesthesiology, National Cancer Center/National Clinical Research Center for Cancer/Cancer Hospital, Chinese Academy of Medical Sciences and Peking Union Medical College, Beijing, 100021 China; 2 Department of Breast Surgery, National Cancer Center/National Clinical Research Center for Cancer/Cancer Hospital, Chinese Academy of Medical Sciences and Peking Union Medical College, Beijing, 100021 China; 3 Department of Anesthesiology, National Cancer Center/National Clinical Research Center for Cancer/Cancer Hospital & Shenzhen Hospital, Chinese Academy of Medical Sciences and Peking Union Medical College, Shenzhen, 518116 China

**Keywords:** Local infiltration analgesia, Ropivacaine, Drainage exit site, Postoperative pain, Breast cancer

## Abstract

**Background:**

Postoperative pain after breast cancer surgery remains a major challenge in patient care. Local infiltration analgesia is a standard analgesic technique used for pain relief after surgery. Its application in patients who underwent mastectomy requires more clear elucidation. This study aimed to investigate the effect of ropivacaine infiltration of drainage exit site in ameliorating the postoperative pain after mastectomy.

**Methods:**

A prospective randomized controlled study was conducted in 74 patients who were scheduled for unilateral mastectomy by standardized general anesthesia. Both intervention group and control group were given infiltration of the two entry points of drainage catheters with 10 ml 0.5% ropivacaine (Group A) (*n* = 37) or 10 ml normal saline (Group B) (n = 37). Pain scores were recorded in post-anesthesia care unit (PACU), at 6 h, 12 h, 24 h and 36 h after operation by using a visual analogue scale (VAS). Postoperative nausea and vomiting (PONV) incidence, postoperative analgesic and antiemetic requirements, the incidence of chronic pain, as well as the quality of recovery were recorded.

**Results:**

The patients in Group A showed a significant reduction in postoperative pain in PACU (*p* < 0.0005), at 6 h (*p* < 0.0005), 12 h (*p* < 0.0005), and 24 h after surgery (*p* < 0.05) when compared to those in Group B. There were more postoperative analgesic requirements in Group B (*p* < 0.05). With regard to the quality of recovery, Group A was shown to be much superior over Group B (*p* < 0.05).

**Conclusions:**

Ropivacaine infiltration of the two drainage exit sites decreased the degree of postoperative acute pain after mastectomy, and this approach improved patients’ quality of recovery.

**Trial registration:**

retrospectively registered in Chictr.org.cn registry system on 24 February 2020 (ChiCTR2000030139).

## Background

Postoperative pain is one of the most common challenges in women following breast cancer surgeries, which impairs rehabilitation and increases the length of hospital stay. About 50% of patients who receive mastectomy might experience persistent postoperative pain [1, 2]. Sensory disturbances such as burning or sensory loss caused from the wound are commonly observed as sequelae of mastectomy, and this might be due to intraoperative nerve injury [3]. However, complaints of acute postoperative pain in patients who received mastectomy are also frequently observed. After mastectomy, a drainage tube is routinely placed, which assists in monitoring bleeding, and fluid or air removal. The wound is infiltrated or irrigated with local anesthetic to reduce acute postoperative pain, and is widely used in surgeries [4, 5]. However, few studies have paid much attention in investigating whether postoperative pain could be effectively alleviated by infiltrating anesthesia at the drainage exit site after mastectomy. Based on our clinical experiences, the major location of the acute pain might be at the insertion sites of the two drainage catheters, which were placed below the skin flap at the end of the surgical procedure. For the firm anchorage of the drain to the skin and sealing of the space around the drain, the two entry points of catheter insertion were chosen over healthy skin below the inframammary fold of the breast, wherein most of the subcutaneous nerves were not damaged, meaning that they were still sensitive to pain. Park’s study [6] reported that surgical drains are associated with high postoperative opioid use after breast cancer surgery, and this supported our observation.

Although multimodal analgesic strategies including opioids, acetaminophen, non-steroidal anti-inflammatory drugs (NSAIDs), peripheral regional techniques, patient-controlled modalities as well as local anesthetic techniques such as wound infiltration are available, postoperative pain still has been poorly managed. Analgesia-related side-effects such as nausea and vomiting, dizziness, constipation and itching are commonly observed, impairing patients’ satisfaction and delaying their discharge time [7, 8]. Postoperative pain after breast cancer surgery can be effectively alleviated by regional nerve block techniques, for example the thoracic paravertebral nerve block (PVB) [9]. But a long learning cycle and invasive nature of the method limited the implementation of PVB in breast cancer surgery.

This prospective randomized controlled study aimed to investigate if infiltration of ropivacaine at the insertion sites of the two drain catheters in mastectomy would reduce postoperative acute pain, postoperative nausea and vomiting (PONV), and chronic pain.

## Methods

### Study design

This prospective, randomized controlled trial was designed in adherence to the CONSORT guidelines and was registered in Chictr.org.cn registry system on 24 February 2020 (ChiCTR2000030139). This study was conducted in Cancer Hospital, Chinese Academy of Medical Sciences between September and November 2019, and has been approved by the institutional ethics committee (IRB Approval Number: 20/351–2135). All patients were followed up until 3 months after discharge from the hospital.

### Participants

Patients who underwent unilateral mastectomy with axillary lymph node dissection (ALND) or sentinel lymph node biopsy (SLNB) were enrolled in this study. Written informed consent was obtained from patients. The patients aged 20 ~ 70 years, and with the American Society of Anesthesiologists physical status of I to III were included. Patients with the following conditions were excluded from the study: history of severe cardiovascular or pulmonary, hepatic, renal, neurologic, and psychiatric or metabolic diseases; history of allergy to any of the potential study medications; active drug abuse; intake of NSAIDs, opioids, or other analgesics in the 24 h before surgery; pregnancy; breastfeeding, active menstruation.

### Randomization

Prior to study initiation, 80 sequentially numbered envelopes containing the allocation were prepared. The involved patients were randomly assigned to 10 ml 0.5% ropivacaine infiltration (Group A) or 10 ml normal saline infiltration (Group B) groups. A physician independent of the study randomly inserted 40 anesthesia strategies for each group into the envelopes. The random allocation sequence was generated using computer-generated random numbers. The researchers opened the envelope to determine as to which anesthesia strategy to implement before the induction of general anesthesia. All perioperative data were collected by an investigator who was blinded to the patient’s allocation, and was responsible for measuring the outcome.

### Interventions

Standard general anesthesia was induced using sufentanil 0.3 ~ 0.6 μg/kg, propofol 1 ~ 2 mg/kg and cis-Atracurium 0.2 ~ 0.4 mg/kg in the two groups. After laryngeal mask airway insertion, the patients were mechanically ventilated to maintain the end-tidal carbon dioxide concentration at 35 ~ 45 mmHg with a fresh gas flow of 2 L/min 60% oxygen. Anesthesia was maintained by constant inhalation of 1.5 ~ 2.5% sevoflurane and constant infusion of remifentanil at a rate of 0.1 ~ 0.2 μg/kg/min. Sufentanil 0.1 μg/kg was added intraoperatively as required. At the end of the surgery all patients received 100 mg of flurbiprofen (an NSAID). Dexamethasone 8 mg (given after induction) and ondansetron 4 mg (given at the end of surgery) were used for prevention of postoperative nausea and vomiting (PONV). All surgical procedures were finished by the same surgical team with the same standardized technique. The two drainage catheters were placed by the surgeon before closing the surgical incision. Before the placement, the subcutaneous tissue of the two entry points of the catheters received local infiltration, which included intervention group (Group A) by 10 ml 0.5% ropivacaine and control group (Group B) by 10 ml normal saline (5 ml for each point). After operation, all patients were extubated and transferred to the post-anesthesia care unit (PACU). Flurbiprofen 100 mg was provided by intravenous injection daily to control the postoperative pain within 3 days after operation. If the pain visual analogue scale (VAS) was ≥3, 100 mg tramadol was administered as a rescue analgesic. For postoperative antiemetic treatment, metoclopramide was intravenously administrated if the nausea VAS was ≥5 or episodes of vomiting ≥2. All patients received standard postoperative therapies according to the pathological characteristics.

### Outcomes

#### The primary outcomes

The pain was immediately assessed after returning to PACU and at 6 h, 12 h, 24 h, and 36 h after operation using a VAS (0 = no pain to 10 = most severe pain). The incidence of PONV was recorded simultaneously, using a three-point ordinal scale (0 = none, 1 = nausea, 2 = retching, 3 = vomiting), and nausea was evaluated by VAS. The number of patients who received postoperative analgesic or antiemetic drugs was recorded.

#### Secondary outcomes

The status of chronic pain in patients was collected using the breast cancer pain questionnaire, which was first developed by Gartner et al. [3]. A Pain Burden Index (PBI) can be calculated according to the data collected by the questionnaire surveys. It was calculated by adding the pain severity scale (0 ~ 10) from anatomic locations of breast, axilla, chest wall and arm, and multiplied by the frequency of pain at each site (constantly—5 points, daily—4 points, occasionally—3 points, weekly—2 points, monthly—1 point, and never—0 points).

The quality of recovery including 40 questions (QoR-40), which is used as a measure of quality of recovery, was distributed to patients for collecting data 24 h after operation. This included five factors of emotional state, physical comfort, psychological support, physical independence, and pain. In all, the highest score was 200 while the lowest being 40 and the more score, the better the results.

Also the remaining data, including the consumption of sufentanil during the surgery, types of surgical procedures, operation and anesthesia time, age, body mass index (BMI), history of smoking and PONV were collected,

### Sample size and statistical analysis

The sample size was calculated based on our preliminary experiment that enrolled 10 cases in each group. The mean pain VAS at 12 h after the surgery was 1.2 ± 2.1 for Group A and 2.9 ± 2.2 for Group B. Using standard sample size calculation formula to achieve a power of 0.8 at α = 0.05, there should be at least 29 patients included in each group to detect a significant difference. Considering the possibility of data censored, a total of 40 patients in each group were recruited to guarantee the sample size. SPSS 23.0 for windows (SPSS, Inc., Chicago, IL, USA) was used for data analysis. Normally distributed continuous data were expressed as means (SD), and were analyzed using analysis of variance (ANOVA), independent-sample t-test or paired t-test. Nonparametric data were analyzed by Mann-Whitney and Wilcoxon text. Two-sided tests were performed to declare statistical significance at *p* < 0.05.

## Results

**Fig. 1:**
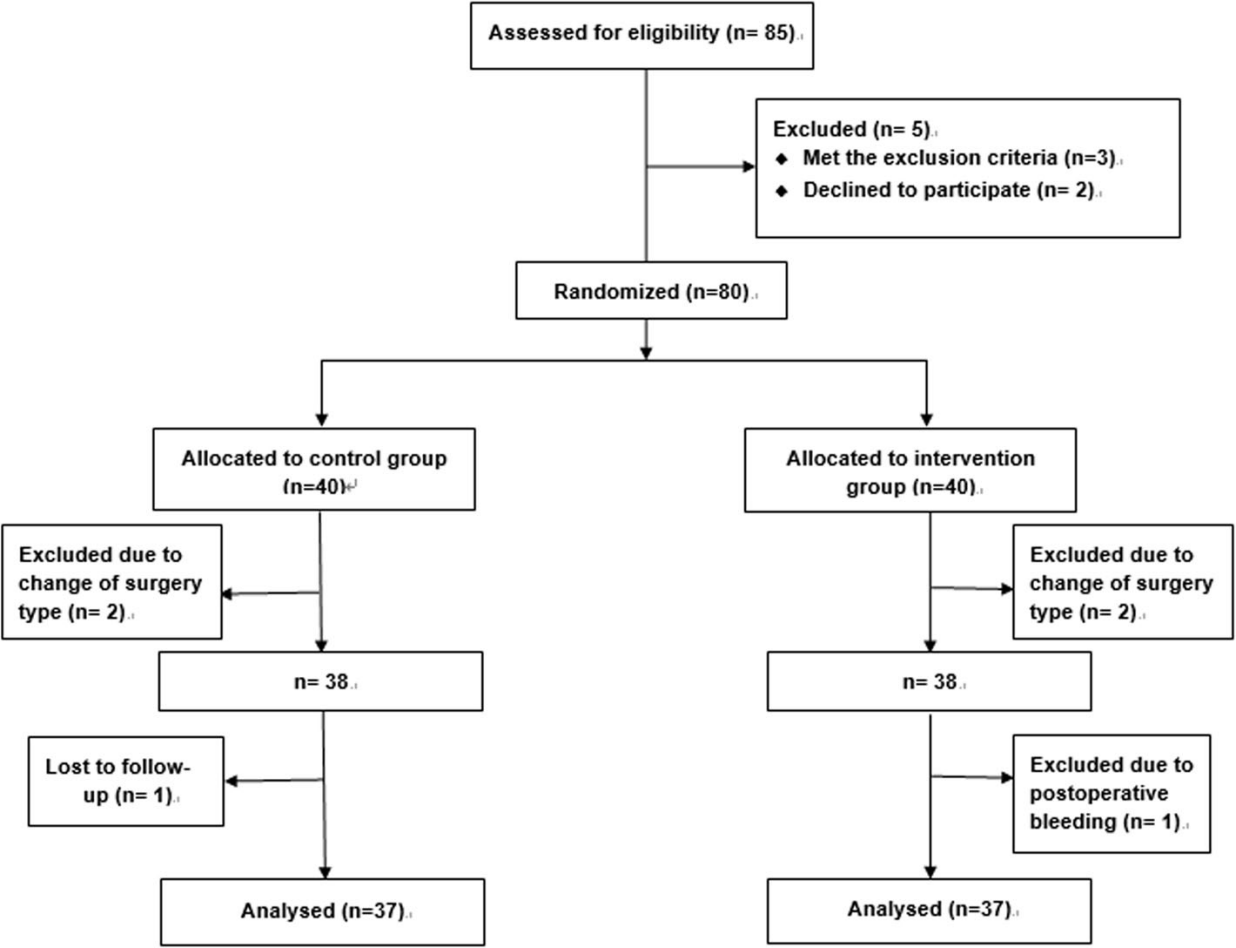
Flow of patients throughout study

The random assignment of the participants into the two groups and analysis of the outcome was presented in Fig. 1. Finally, a total of 74 patients were included in this study. Demographic characteristics, including age, body weight, body height, BMI, smoking status, and history of PONV were comparable in both groups (Table 1). In addition, no significant differences were found in the consumption of sufentanil during surgery, the durations of anesthesia and surgery, and the types of surgical procedures used (Table 1).

**Table 1:** Baseline characteristics of patients in the study and the control groups

	Group A(***n*** = 37)	Group B(***n*** = 37)	***P*** value
**Age (years)**^**a**^	52.1 ± 9.0	49.1 ± 9.9	0.17
**Weight (kg)**^**a**^	61.8 ± 8.7	59.8 ± 8.4	0.32
**Height (cm)**^**a**^	160.3 ± 5.5	159.4 ± 5.0	0.44
**Body mass index**^**a**^	24.0 ± 3.6	23.9 ± 3.1	0.82
**Smoking (%)**	0	0	–
**History of PONV (%)**	5 (13.5%)	4 (10.8%)	0.48
**Surgical procedure**			0.41
mastectomy + axillary dissection (%)	30 (80.1%)	27 (73.0%)	
mastectomy + SNLB (%)	7 (19.9%)	10 (27.0%)	
**Surgery time (min)**^**a**^	98.1 ± 31.3	111.0 ± 28.8	0.07
**Length of anesthesia (min)**^**a**^	113.5 ± 30.1	125.5 ± 27.9	0.08
**Consumption of Sufentanil (μg)**^**a**^	24.8 ± 5.7	23.8 ± 5.0	0.42

^a^Values are expressed as the mean ± standard deviation; *PONV* postoperative nausea and vomiting; *SNLB* sentinel lymph node biopsy

**Fig. 2:**
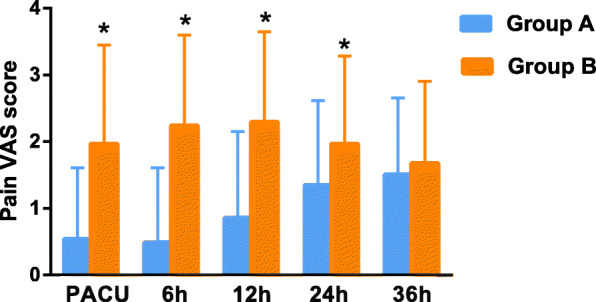
Pain VAS score of patients in the study and control groups. Compared with Group B, postoperative pain in Group A was significantly reduced in PACU (VAS score, 0.54 ± 1.07 vs. 1.97 ± 1.48, *p* < 0.0005), at 6 h (VAS score, 0.49 ± 1.12 vs. 2.24 ± 1.36, *p* < 0.0005), 12 h (VAS score, 0.86 ± 1.29 vs. 2.30 ± 1.35, *p* < 0.0005), and 24 h after operation (VAS score, 1.35 ± 1.27 vs. 1.97 ± 1.32, *p* < 0.05). *p < 0.05; Group A: intervention group (ropivacaine infiltration); Group B: control group (normal saline infiltration); PACU: Post-anesthesia care unit; VAS: Visual analogue scale

Surgical drains were reported to be associated with high postoperative opioid use after breast-conserving surgery^6^. Although whether surgical drains increase opioid consumption after mastectomy has not been investigated, pain originating from the insertion sites of the two drainage catheters constitutes a major part of acute postoperative pain. In this study, we found that ropivacaine infiltration of the two drainage exit sites have significantly reduced the postoperative pain in PACU (VAS score, 0.54 ± 1.07 vs. 1.97 ± 1.48, *p* < 0.0005), at 6 h (VAS score, 0.49 ± 1.12 vs. 2.24 ± 1.36, *p* < 0.0005), 12 h (VAS score, 0.86 ± 1.29 vs. 2.30 ± 1.35, *p* < 0.0005), and 24 h after operation (VAS score, 1.35 ± 1.27 vs. 1.97 ± 1.32, *p* < 0.05) (Fig. 2). However, at 36 h after operation, a significant difference was not observed any more (1.51 ± 1.15 vs. 1.68 ± 1.23, *p* > 0.05) (Fig. 2). More number of patients required for postoperative rescue analgesic in Group B than in Group A (17 vs. 7, *p* < 0.05).

We did not collect pain scores of restricted movement of the shoulder and values of arm abduction angle. Because the operated arm was tightly bound and forbidden to move by surgeons within 3 days after the operations, in case of wound dehiscence, subcutaneous effusion and hematoma.

The intervention showed no effect on the incidence of chronic pain within 3 months, and PBI in the two groups showed similar results (Table 2). No significant differences were found between the two groups in terms of PONV incidence (Table 3) and the requirements for postoperative antiemetic treatment. The QoR-40 score of Group A was significantly higher than Group B (185.8 ± 8.3 vs 179.7 ± 11.2, *p* < 0.05).

**Table 2:** Incidence of chronic pain in the study and the control groups

		Group A (***n*** = 37)	Group B (***n*** = 37)	***p*** value
**Location**				
**Chest wall**		8 (21.6%)	12 (32.4%)	0.30
	**Axillary**	9 (24.3%)	7 (18.9%)	0.58
	**arm**	5 (13.5%)	4 (10.8%)	0.72
**Total**		19 (51.4%)	16 (43.2%)	0.49
**PBI**^a^		7.3 ± 9.7	7.3 ± 9.0	0.75

^a^Values are expressed as the mean ± standard deviation; *PBI* Pain Burden Index

**Table 3:** Incidence of PONV in the study and the control groups

	Group A (***n*** = 37)	Group B (***n*** = 37)	***p*** value
**In the PACU**			
PONV	9 (24.3%)	11 (29.7%)	0.93
Asymptomic	28 (75.7%)	26 (70.3%)	
**PACU-6 h**			
PONV	8 (21.6%)	8 (21.6%)	0.90
Asymptomic	29 (78.4%)	29 (78.4%)	
**6 h–12 h**			
PONV	6 (16.2%)	12 (32.4%)	0.12
Asymptomic	31 (83.8%)	25 (67.6%)	
**12 h–24 h**			
PONV	3 (8.1%)	5 (13.5%)	0.46
Asymptomic	34 (91.9%)	32 (86.5%)	
**24 h–36 h**			
PONV	0 (0%)	1 (2.7%)	0.31
Asymptomic	37 (100%)	36 (97.3%)	

*PONV* postoperative nausea and vomiting; *PACU* post-anesthesia care unit

## Discussion

To our knowledge, this is the first prospective randomized controlled study to reveal ropivacaine infiltration of the two drainage exit sites, which significantly reduced postoperative pain and analgesic requirements and improved the quality of recovery after mastectomy.

Park’s study [10] reported that compared with other breast surgical procedure types, mastectomy required consumption of more opioids during the first week postoperatively. Opioid consumption plays an important role in postoperative pain control after mastectomy. However, opioid-related adverse effects led to other problems that delay the recovery, and so novel analgesics or strategies with less side-effects are urgently needed. Kairaluoma et al. [11] have found that PVB could improve postoperative pain and reduce opioid consumption after modified radical mastectomy. Nevertheless, compared with local infiltration anesthesia, the regional block technique is considered more challenging technically, and needs a longer learning period. According to our results, ropivacaine infiltration of the two drainage exit sites during mastectomy is a simple, easy, and economical approach for pain relieving, without any opioid-related adverse effects. Correspondingly, postoperative analgesic requirements were reduced, and the quality of recovery was improved.

To compare with the previous similar studies, in Table 4 we summarized 8 randomized controlled trials that evaluated the efficacy of local analgesic for pain relief in breast cancer surgery, in which the local analgesic was either injected into the wound preoperatively or instilled through the drainage tube into the wound postoperatively. However, in this study, we chose to perform local infiltration anesthesia of the two drainage exit sites with ropivacaine, based on the evidence that pain caused by the drainage plays a vital role in postoperative pain. Five of the above mentioned trials [12–16] showed no differences between the control and experimental groups, in which Baudry et al.’s studies [12] enrolled patients who received breast-conserving surgery with or without ALND. However, the postoperative pain of different types of surgical technique remained different. The breast-conserving surgeries are susceptible to acute pain of the wound. In contrast, mastectomy surgeries are susceptible to burning, sensory loss or other abnormal sensations due to nerve damage, while acute pain is not reported as the main complaint [20]. Thus, it is not precise to compare the pain score between these two types of surgery due to the different mechanisms of pain. Our study is designed by including patients who received mastectomy, which provided comparable results. Nirmala et al. [17–19] have found that the local analgesic group showed significant reduction in the postoperative pain within 90 min, 6 h, and 15 h, respectively. Although the infiltration location in our study was different from theirs, local infiltration anesthesia showed effective results for patients who received mastectomy, and had a longer effective duration (24 h).

**Table 4:** Characteristics of the selected randomized controlled trials

Study	Research aim	Surgical technique	Intervention	Infiltration locations	Result	Ref
Baudry [2008]	evaluate the effect of R wound infiltration	MRM or partial mastectomy with ALND	R: 4.75 mg/mL R 40 mL C: NS 40 mL	The wound	no differences	[12]
Johansson [2003]	whether infiltration with R + fentanyl improves PP	Partial mastectomy with or without ALND	R1: 0.375% R R2: 0.375% R + Fentanyl 0.5 μg/kg C: Nil	The wound	no differences	[13]
Johansson [2000]	whether infiltration with R improves PP	Partial mastectomy with or without ALND	R: R 3.75 mg/mL C: NS 0.3 mL/kg	The wound of breast and axilla	no differences	[14]
Rica [2007]	if infiltration with R could improve PP	Mastectomy and ALND	R1: Preoperative 0.2% R 20 mL + NS to 80 mL R2: Postoperative 0.2% R 20 mL + NS to 80 mL	The wound	no differences	[15]
Talbot [2004]	determine the influence of B irrigation on PP	MRM	B: 0.5% B 20 mL C: NS	Through the axillary drain into the axillary wound	no differences	[16]
Nirmala [2019]	Whether wound instillation with B improve PP	MRM	R: 0.25% B 40 ml C: 40 ml normal saline	through chest and axillary drains into the wound	providing better analgesia within 15 h	[17]
Vigneau [2011]	document the effect of R infiltration	Mastectomy or lumpectomy with ALND	R: R 7.5 mg/mL solution 20 mL C: NS 20 mL	The wound	PP was lower at 2, 4 and 6 h after surgery	[18]
Albi-Feldzer [2013]	evaluate the influence of R wound infiltration	Conservative surgery with ALND, MRM with or without ALND	R: 0.375% R 3 mg/kg mixed with saline C: Saline solution	the wound, the 2nd & 3rd intercostal spaces and the humeral insertion of major pectoralis	decreased immediate PP (≤90 min)	[19]

*ALND* axillary lymph node dissection; *B* bupivacaine; *C* control; *MRM* modified radical mastectomy; *NS* normal saline; *R* ropivacaine; *PP* postoperative pain

The incidence of PONV in the two groups remained similar. However, from 6 h to 12 h after operation, PONV occurred in 6 patients in Group A and 12 in Group B. This difference might due to the side-effects of higher tramadol requirement in Group B. At 3-months follow-up, no significant differences were discovered with regard to the incidence of chronic pain. The mechanism of chronic pain is complicated, and it is still poorly controlled. Although our intervention decreased the postoperative pain more effectively, no surgical, demographic, and psychosocial factors that influenced chronic pain after breast surgery considered [21]. It has been reported that 30% ~ 51% of patients suffered from persistent pain after breast cancer surgery [2]. In our study, 51.35% of patients in Group A and 43.24% in Group B suffered from chronic pain. Nearly half of the patients are tortured by chronic pain. Therefore, it is still regarded as a great challenge to explore the reduction and treatment of chronic pain after breast cancer surgery. Furthermore, combined with the other pain-control methods, local infiltration anesthesia might further reduce the occurrence of postoperative acute pain, and even have positive effects on chronic pain.

However, there are certain limitations in this study that require consideration. Firstly, the sample size is small, and so it is not sufficient to perform subgroup analysis. Secondly, follow-up of the patient’s pain after 3-months was not evaluated.

## Conclusions

In conclusion, ropivacaine infiltration of two drainage exit sites effectively decreased the degree of postoperative acute pain and analgesic requirements within 24 h, and meanwhile improved patients’ quality of recovery. Further large scale studies are warranted to study the outcomes in the future, and explore efficient approach that relieve pain after mastectomy in long run.

## Data Availability

All the data used and analyzed are available from corresponding authors upon the reasonable request.

## Abbreviations

PACU: Post-anesthesia care unit
PONV: Postoperative nausea and vomiting
VAS: Visual analogue scale
NSAIDs: Nonsteroidal anti-inflammatory drugs
BMI: Body mass index
SD: Standard deviation
ANOVA: Analysis of variance
PBI: Pain burden index
PVB: Paravertebral nerve block
ALND: Axillary lymph node dissection
SLNB: Sentinel lymph node biopsy
